# Diurnal Emissions of Sea Spray Aerosols in Algal Blooms

**DOI:** 10.1021/acs.est.5c12650

**Published:** 2025-11-03

**Authors:** J. Michel Flores, Miri Trainic, Daniella Schatz, Ilan Koren, Assaf Vardi

**Affiliations:** † Department of Earth and Planetary Science, 34976 Weizmann Institute of Science, Rehovot 7610001, Israel; ‡ Department of Plant and Environmental Sciences, 34976 Weizmann Institute of Science, Rehovot 7610001, Israel

**Keywords:** sea spray aerosol, diurnal emission, *Gephyrocapsa huxleyi* coccolithophores, algal blooms

## Abstract

Understanding the factors that influence the production of sea spray aerosols (SSA) is crucial for better understanding their impact on our climate. However, the role of oceanic microbial activity in contributing to SSA production is still underexplored. Here, we investigated the dynamics of SSA number concentration (*N*_SSA_) during induced algal blooms within three airtight mesocosm enclosures. Monitoring algal abundance throughout a 24-day experiment revealed two main blooms: a mixed algal bloom followed by an extensive bloom of the coccolithophore *Gephyrocapsa huxleyi* and its subsequent demise. We observed two main patterns in *N*_SSA_: (1) a diurnal variation with higher daytime emissions; and (2) a progressive decrease in *N*_SSA_ throughout the bloom succession, with peak *N*_SSA_ during the first mixed algal bloom and a decline during the *G. huxleyi* bloom and its demise. We hypothesize that photosynthetic processes could contribute to the observed diurnal changes, potentially through effects of enhanced bubble formation and subsequent SSA production. The suppression of *N*_SSA_ during the *G. huxleyi* bloom and its demise correlates with the accumulation of particulate organic carbon and transparent exopolymer particles, which can act as surfactants and potentially suppress SSA production by altering surface tension dynamics. Our findings underscore the complex interplay between algal bloom dynamics and SSA production, with implications for understanding aerosol dynamics in marine environments, particularly under changing climate conditions.

## Graphical abstract

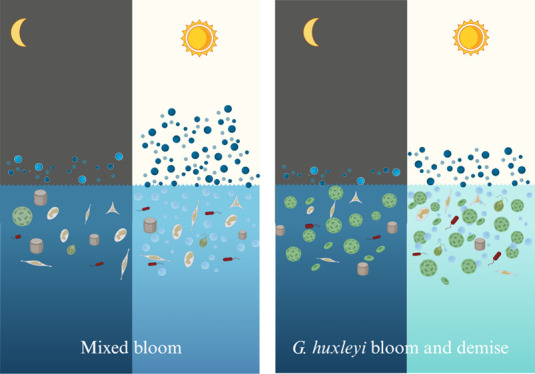

## Introduction

Sea spray aerosols (SSA), originating from the ocean’s surface, play a key role in atmospheric chemistry, Earth’s radiation balance, cloud formation, precipitation properties, and marine ecology.^1–7^ Our current understanding indicates that SSA forms either by the mechanical tearing from the waves’ crest at high wind speeds or when wind stress causes waves to break, entraining air into the ocean’s surface and creating bubbles that burst, resulting in fine droplets (film and jet droplets).^8–10^ Significant efforts have been made to assess the quantity, size, and composition of the film and jet droplets’ dependence on the physical and chemical properties and biological activity^4,11–19^ of the ocean surface. Despite these substantial research efforts, parametrizations of sea spray production still have large uncertainties.^20,21^ While there is considerable understanding of the dependence of SSA properties and flux on physical properties (e.g., wind speed), the direct, mechanistic interplay between microbial activity at the ocean surface and SSA formation and emission is not fully understood.^17^

One crucial variable in this context is SSA concentration (*N*_SSA_). *N*_SSA_ has been shown to have a nonlinear positive relationship with wind speed,^15,19,22–25^ to depend on sea surface temperature,^15,18,26–31^ wave state,^28,32^ salinity,^33,34^ and surface-active organic species or surfactants.^30,31,33,35–38^ A special class of surface-active material are transparent exopolymer particles (TEPs).^39^ TEPs are gel-like surface-active particles that are secreted by many marine microorganisms.^40^ TEPs that are not attached to sufficiently dense material can rise to the surface in the open ocean to form or stabilize the sea surface microlayer^41^ possibly affecting the concentration and composition of SSA.^42^ Studies examining the relationship between microbial activity and SSA production have yielded conflicting results. For example, in laboratory settings, SSA production increased when diatomaceous exudates^12^ or actively growing bacteria and phytoplankton^43^ were present in the seawater, while a decrease in production occurred in the presence of surfactants,^35^ or with an increase in algal biomass.^26^ Similarly, studies using artificial bubbling experiments at sea have found mixed results. Some studies report positive linear correlations between nanophytoplankton cell abundances and *N*_SSA_.^37,38^ Others, an increase in the number and mass of SSA during the day, where chlorophyll-*a* concentrations were greater than ∼2 mg m^–3^.^13,14^ In contrast, another study found decreasing *N*_SSA_ in daytime and during phytoplankton activity.^44^ A satellite observational study reported a negative correlation between chlorophyll-a concentration and coarse-mode aerosol optical depth in the South Pacific Gyre on a seasonal time scale, suggesting that higher algal densities may suppress particle production or emission.^45^ A large-scale in situ study conducted over thousands of kilometers in the open ocean in the Pacific and Atlantic Oceans found a diurnal cycle of *N*_SSA_ with higher *N*_SSA_ during the daytime in oligotrophic waters.^15^ Oligotrophic waters are usually characterized by low biomass and low chlorophyll, while temperature has been shown to drive SSA production in these regions,^18^ biological influences on *N*_SSA_ remain largely unassessed. The *N*_SSA_ diurnal cycle had no significant links with daily variations in surface winds, atmospheric conditions, radiation, pollution, or oceanic properties. Only the daily mean sea surface temperature positively correlated with the day-to-night increase in *N*_SSA_. It is, therefore, clear that the bulk effect of the ocean’s biological component on the emitted *N*_SSA_ has not yet been resolved.

To better understand the effect of phytoplankton activity on the emitted *N*_SSA_, we used an induced algal blooms mesocosm experiment^46–48^ to mimic as closely as possible the complexity of a natural bloom. In this experiment, we followed the dynamics of the cosmopolitan phytoplankton *Gephyrocapsa huxleyi* ((Lohmann) P.Reinhardt previously known as *Emiliania huxleyi*). *G. huxleyi* is a bloom-forming phytoplankton species^49^ that forms annual blooms that can span thousands of square kilometres. *G. huxleyi* blooms evolve along three general phases: initiation, exponential growth and demise.^50^ The demise phase is primarily attributed to virus-induced mortality driven by the *G. huxleyi*-specific virus (EhV).^51,52^ This mesocosm experiment focused on assessing the impact of viral infection on algal bloom dynamics and has yielded findings on the metabolic footprint on the dissolved organic matter after viral infection,^53^ on the composition of the bloom-associated microbiome,^46^ and dimethylsulfide emission.^54^

To follow the SSA emission throughout the different phases of the *G. huxleyi* bloom, three large airtight covered enclosures (Bags 5, 6, and 7; we are maintaining the same numbering as^46^ and^53^ for consistency between manuscripts; see Figure 1) were immersed in a fjord near Bergen, Norway, and filled with 11,000 L of fjord water containing natural planktonic communities, nutrients were added on a series of consecutive days (see Mesocosm setup below and Vincent et al.^46^). We combined the daily monitoring of various biological and biogeochemical parameters with measurements of the SSA emitted by artificial bubbling and quantified the impact of the different bloom stages on *N*_SSA_. Our results show a clear and repeatable diurnal cycle of *N*_SSA_ concomitant to a suppression of *N*_SSA_ throughout the bloom succession, with *N*_SSA_ peaking during an initial mixed bloom and diminishing during the *G. huxleyi* bloom.

**Figure 1:**
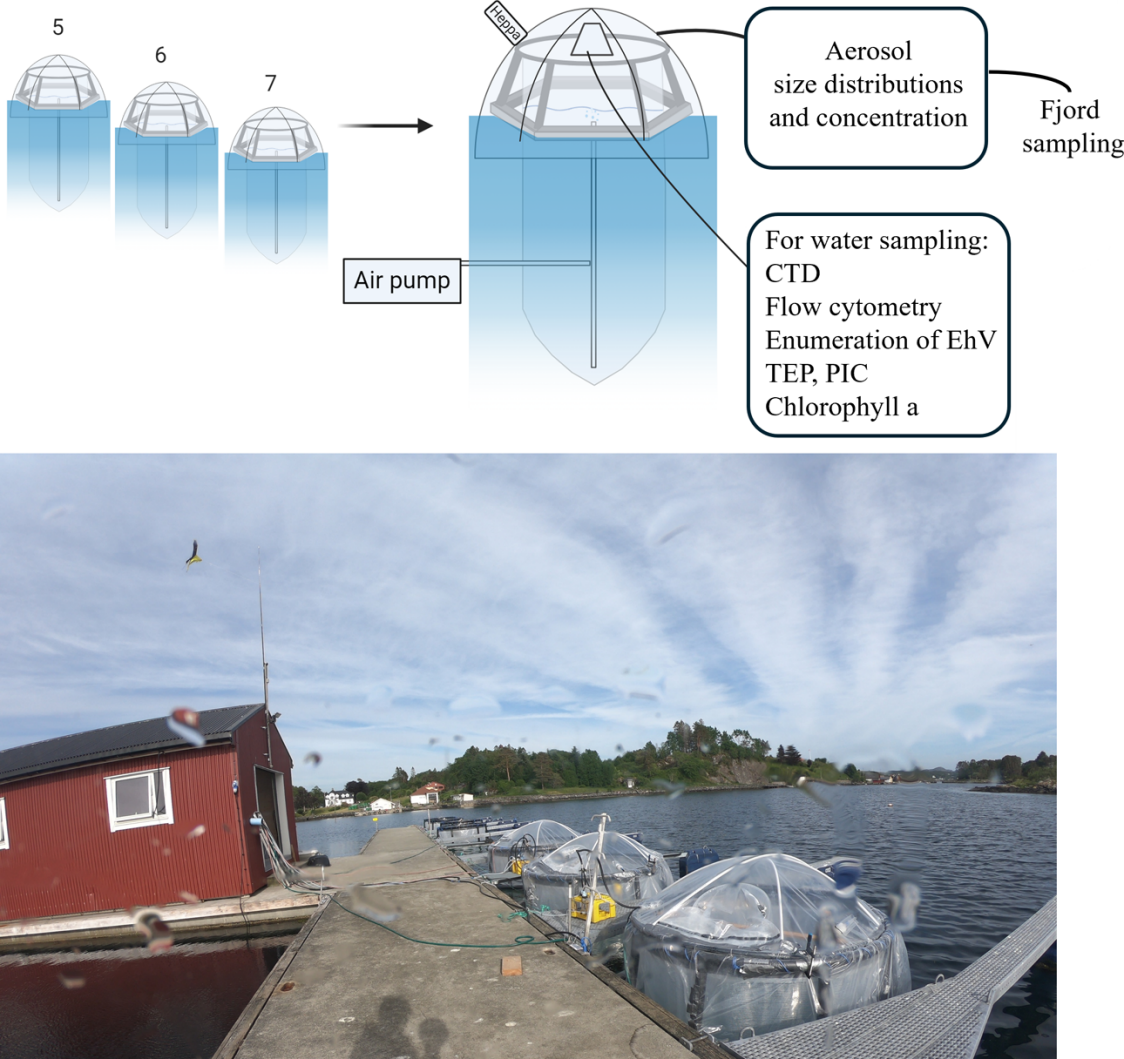
Overview of the mesocosm setup in the marine biological station Espegrend, Norway (60°16′11N; 5°13′07E). The experimental setup consisted of three transparent enclosure bags mounted on floating frames, sealed with an airtight cover, and moored to a raft in Raunefjord. The bags were filled with surrounding fjord water 1 day before the experiment began and supplemented with nutrients throughout. The schematic (created with BioRender.com) illustrates the connection between the bags and the aerosol size and concentration measurement system (SMPS-APS) located in a nearby hut. Water sampling was conducted daily between 7:00 and 9:00 AM through the trapezoid door, while samples for flow cytometry were collected throughout the day via a small, HEPA-filtered opening.

## Methods

### Mesocosm Setup

The mesocosm experiment AQUACOSM VIMS-Ehux was carried out between 24th May (day 0) and 16th June (day 23) 2018 in Raunefjorden at the University of Bergen’s Marine Biological Station Espegrend, Norway (60°16′11 N; 5°13′07E; see Figure 1). The experiment consisted of seven enclosure bags made of transparent polyethylene (11 m^3^, 4 m deep and 2 m wide, permeable to 90% photosynthetically active radiation) mounted on floating frames and moored to a raft in the middle of the fjord. The bags were filled with surrounding fjord water (day – 0; pumped from 5 m depth) and continuously mixed by aeration. Each bag was supplemented with nutrients at a nitrogen-to-phosphorus ratio of 16:1 according to the optimal Redfield Ratio (1.6 μM NaNO_3_ and 0.1 μM KH_2_PO_4_ final concentration) on days 0–5 and 14–17. In contrast, on days 6, 7, and 13, only nitrogen was added to limit the growth of picoeukaryotes and favor the growth of *G. huxleyi*, which is more resistant to phosphate-limited conditions.^55^ Silica was not added as a nutrient source to suppress the growth of diatoms and to enhance *G. huxleyi* proliferation. Bags 1- 4 were kept uncovered and sampled to explore microbial interaction (see e.g., refs 46,53 ). Bags 5, 6, 7 were covered to measure and collect aerosols. The bags were opened to sample the water every morning between 07:00 and 09:00.

### Aerosol Instrumentation

Two aerosol instruments were installed next to the covered bags (Figure 1; in the red cabin): a scanning mobility particle sizer (SMPS, TSI Inc., Minnesota, USA) for size distribution measurements from 0.013 to 0.737 μm, and an aerodynamic particle sizer (APS; TSI Inc., Minnesota, USA) for size distribution measurements from 0.523 to 20 μm. A diffusion dryer was installed before the SMPS and APS, which reduced the sampled air’s relative humidity to below 30%. The flow through the SMPS–APS system was ∼5.3 L min^–1^ (lpm). The SMPS and APS were set to do a full scan of the particle distribution every 5 min, and both instruments were set to measure continuously throughout the experiment and were time-synchronized with the local date and time. Given that only one SMPS-APS system was available, it was connected to one bag at a time using conductive tubing of 1.9 cm inner diameter. Before the experiment started and on days 3, 9, 10, and 22, the fjord’s air was measured for comparison (Figure 2 shows the example for days 9 and 10).

**Figure 2:**
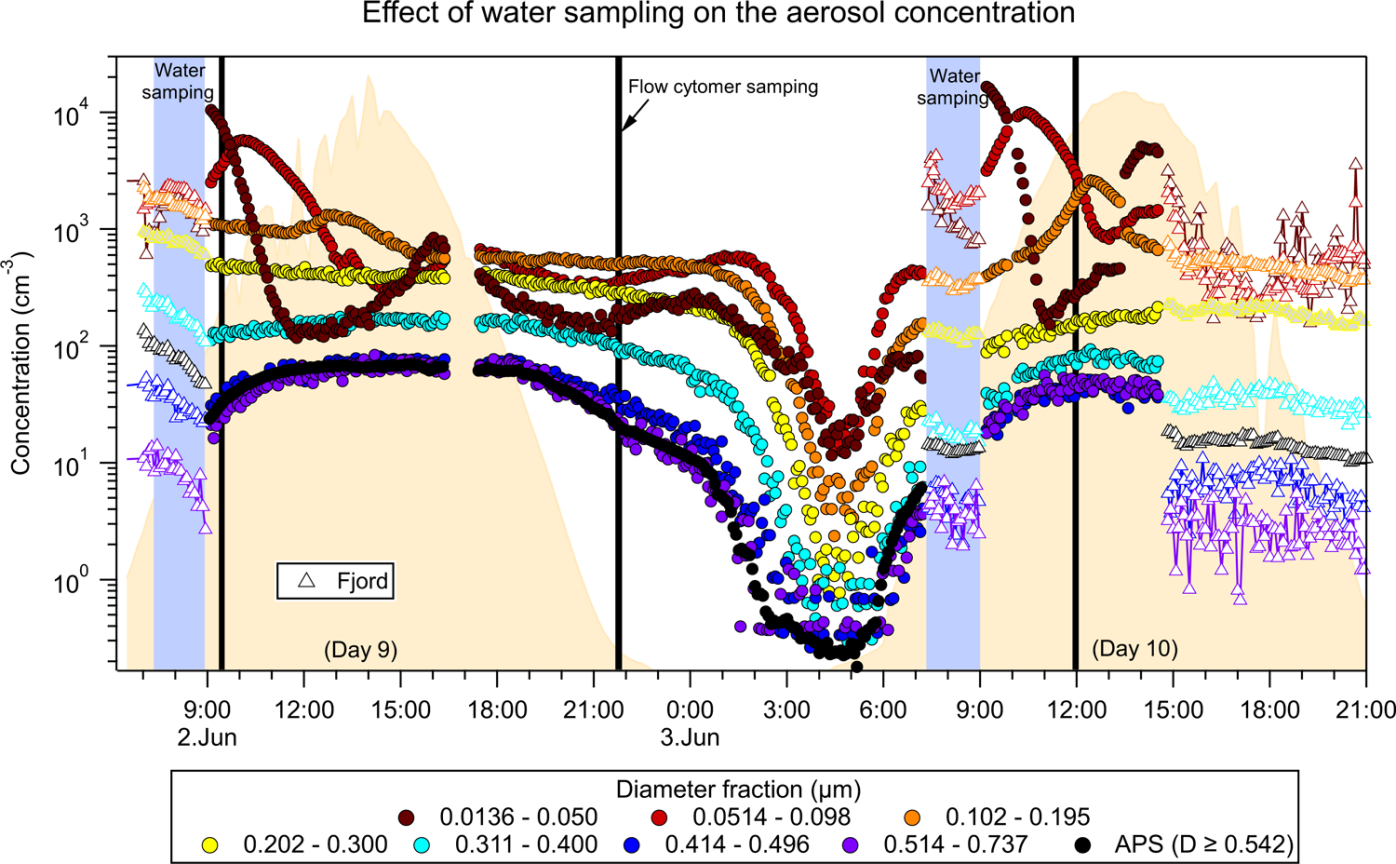
Effect of water sampling on the SSA measurements. Sea spray aerosol concentration for eight different diameter fractions of Bag 6 (colored circle markers) and the fjord (colored triangle markers) during day 9 (June 2, 2018) and day 10 (June 3, 2018) of the experiment. The diameter fractions were calculated using the SMPS data, and the APS concentration is shown for diameters ≥0.542 μm. The time the bag was opened for water sampling is shown with the blue columns, and the times of flow cytometry samplings are shown with vertical black lines.

### Water Sampling

A full description of the water sampling and measurements performed has been published by Kuhlisch et al. and Vincent et al.^46,53^ For the analysis presented in this work, we used the abundance of calcified *G. huxleyi*, the *G. huxleyi* virus (EhV), bacteria, nanophytoplankton, picophytoplankton, and ciliates. Additionally, water temperature and salinity, and the transparent exopolymer particles (TEP), particulate inorganic carbon (PIC), particulate orfanic carbon (POC), and chlorophyll-a concentration data were used. All data were published and available in Vincent et al.^46^

In short, the enumeration of phytoplankton cells was performed using flow cytometry: water samples were collected from ∼1 m depth in 50 mL tubes, prefiltered using 40 μm cell strainers, and immediately analyzed with an Eclipse iCyt (Sony Biotechology, Champaign, IL, USA) flow cytometer. For all other analyses, samples were collected from the same depth in 10- to 20-L carboys (rinsed with <100-kDa filtered seawater) using a peristaltic pump at ∼5 L/min and prefiltered through a 200-μm nylon mesh. Water temperature and salinity were measured in each bag and the surrounding fjord water using a SD204 CTD/STD (SAIV A/S, Laksevag, Norway). Data points were averaged for 1–3 m depth (descending only).

## Results

### Effect of Water Sampling on *N*_SSA_

To monitor the various biological and biogeochemical parameters during the experiment, we opened the airtight bags and sampled the water every morning between 7:00 and 9:00. To check the effect in the aerosol population and possible contamination, we checked the aerosol number concentration for different diameter ranges as a function of time (Figure 2). We found that while diameters larger than 0.2 μm were not affected by the opening of the bags, diameters smaller than 0.2 μm were affected by a significant jump in number concentration. For example, by focusing on the morning of the third of June, 2018 in Figure 2, we can see how diameters in the range between 0.014–0.050 μm had over 2 orders of magnitude jump in concentration from before opening the bag (∼07:30) to after it was closed again (∼09:00). The increase in concentration was followed by a rapid decrease within 2 h. Concomitant to this behavior, the diameters range between 0.051–0.098 μm also had a jump in concentration after the opening of the bag, but they continued to increase in concentration after closing the bag, suggesting growth. We hypothesize this behavior to be related to secondary aerosol formation, due to the significant discrepancies in the aerosol concentration with the air above the fjord, though we cannot confirm it due to the lack of chemical information. We measured the concentration of the air above the fjord while the bags were opened. In the three fjord measurements that can be seen in Figure 2, the aerosol concentration of all the diameter ranges does not follow the concentration trends when the bags are sealed. On the one hand, this suggests that the over two-order-of-magnitude increase in concentration for the 0.014–0.050 μm diameters is not caused by the intrusion of fjord air but rather by secondary aerosol formation processes and that this affected the concentration of up to 0.2 μm in diameter. On the other hand, we can see that above 0.2 μm in diameter, the bags’ opening did not significantly affect the measurements, with all diameter ranges following the same trend before the bags were opened. For the diameter range between 0.102–0.195 μm, there is a significant increase in concentration after closing the bag, which might be due to growth, but given this uncertainty, we excluded it from the analysis. We, therefore, use the SSA number concentration for diameters greater than 0.2 μm (*N*_SSA>0.2μm_) as proxies for the diel cycle to help understand the impact of oceanic variables on the *N*_SSA_ diel cycle. Nevertheless, we acknowledge that a significant fraction of primary SSA can occur below 0.2 μm.

### Diurnal Emissions of SSA and the Effect of Different Phytoplankton Bloom Stages

We explored the temporal pattern of the SSA number concentration for diameters greater than 0.2 μm (*N*_SSA>0.2μm_; Figure 3a. see Effect of water sampling on *N*_SSA_ and Figure 2 for the explanation of why we focused only above *D* ≥ 0.2 μm). We found a diurnal change in *N*_SSA>0.2μm_ in the bloom phases throughout the experiment, with higher concentrations during the day than at night (Figure 3a). *N*_SSA>0.2μm_ increases between 6:00–7:00 am, about an hour and a half after the photosynthetically available radiation (PAR) values began to increase, continuing throughout the morning and midday, and reaching maximum concentrations after the peak PAR (∼14:00). This is followed by a steady decrease in *N*_SSA>0.2μm_ after 15:00, reaching the minimum concentration around 03:00–04:00.

**Figure 3:**
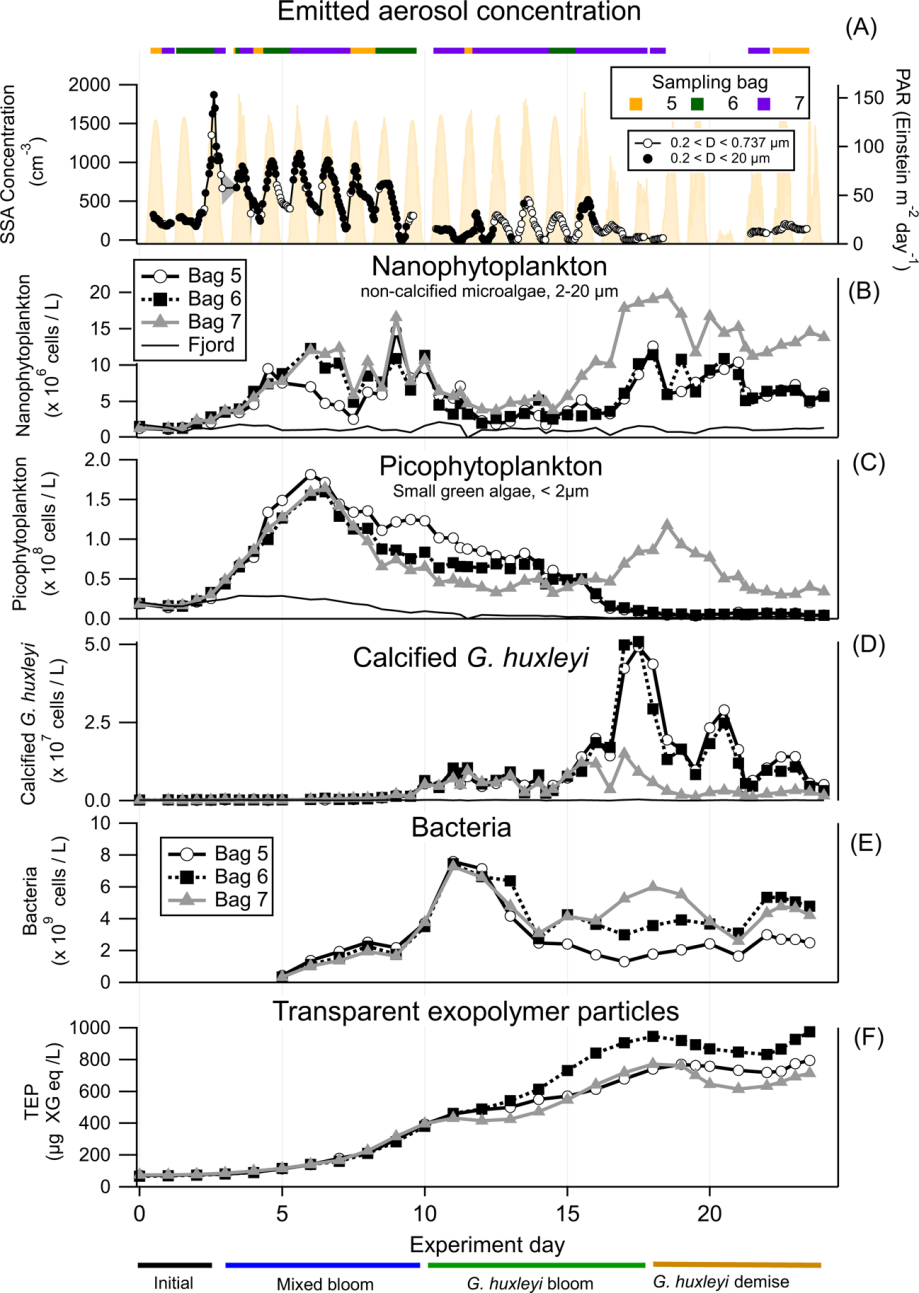
Sea spray aerosol concentration temporal patterns and microbial succession. (A) Sea spray aerosol (SSA) concentration throughout the experiment as measured with the SMPS for diameters larger than 0.2 μm (open black circles) and the APS (filled black circles). The colored bar (orange for Bag 5, dark green for Bag 6, and purple for Bag 7) at the top of the panel indicates when a specific bag was being sampled with the SMPS-APS system. The photosynthetically available radiation (PAR) is shown in light yellow. (B) Nanophytoplankton abundance, including non-calcifying G. huxleyi measured by flow cytometry, based on low side scatter and high chlorophyll signals. (C) Picophytoplankton abundance measured by flow cytometry, based on low side scatter and low chlorophyll signals. (D) Calcified *G. huxleyi* abundance measured by flow cytometry, based on high side scatter and high chlorophyll signals. (E) Absolute abundance of bacteria measured by flow cytometry after SYBR green staining. (F) TEP concentration measured by Alcian blue staining over time. The different bloom stages, as defined by Vincent et al.,^46^ are presented at the bottom.

Concomitantly, we followed the temporal abundance dynamics of nanophytoplankton, picophytoplankton, and specifically the bloom-forming *G. huxleyi* (Figure 3b–d), which were characterized previously.^46,53^ In short, there were four different phases of phytoplankton bloom succession in the water of the three bags: (i) the initial phase (Days 0–2), where the nanophytoplankton, picophytoplankton and the *G. huxleyi* cell concentrations are similar to those in the fjord; (ii) the mixed bloom phase (Day 3–9), which was dominated by dinoflagellates, *Leptocylindrus minimus* and the picophytoplankton *Bathycoccus* and *Micromonas*, and had low calcified *G. huxleyi* cells;^46^ (iii) the *G. huxleyi* bloom (Day 10–17); and (iv) the *G. huxleyi* demise phase (Day 18–23). Bacteria were present throughout the experiment, between day 5 and 11, they had over a 10-fold increase in abundance, while bacteria were less abundant during the *G. huxleyi* bloom and demise compared to the mixed bloom.^46^

The emerging *N*_SSA>0.2μm_ diurnal patterns showed the general trend described above, but with different maxima and minima throughout the various phases (Figures 3 and 4). In all three bags, the highest *N*_SSA>0.2μm_ was observed during the mixed bloom phase compared to the initial phase and the *G. huxleyi* bloom and demise (Figure 4). For Bags 6 and 7, *N*_SSA>0.2μm_ decreased during the *G. huxleyi* bloom phase, while diurnal variations persisted. Only during the *G. huxleyi* demise phase were the diurnal changes diminished. For Bag 5, the diurnal *N*_SSA>0.2μm_ changes disappear during the *G. huxleyi* bloom and demise phases. In bag 7, we observed the highest viral infection of *G. huxleyi*,^46^ but there was no correlation between the level of viral infection and *N*_SSA>0.2μm_.

**Figure 4:**
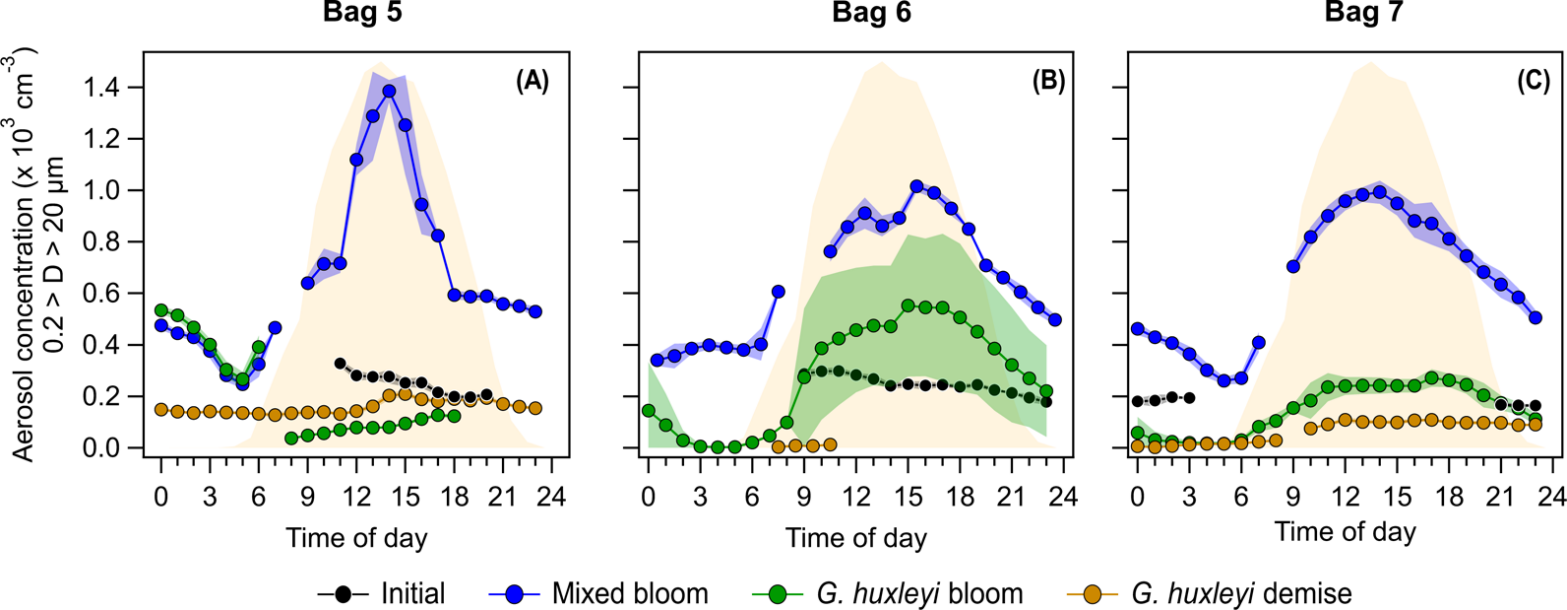
Twenty-four-hour cycle of the *N*_SSA>0.2μm_ for Bags 5, 6, and 7 averaged over the different bloom periods. The emerging diurnal patterns show different maximum and minimum throughout the various phases. In the three bags, the highest *N*_SSA>0.2μm_ was seen during the mixed bloom period compared to the initial phase and the *G. huxleyi* bloom and demise. The averaged photosynthetically available radiation (PAR) throughout the experiment is shown in light yellow. The shaded areas represent one standard deviation of the averaged *N*_SSA>0.2μm_ for each bloom period.

The differences between the maximum (daytime) and minimum (nighttime) *N*_SSA>0.2μm_ varied during the different stages. In the initial phase, the maximum daytime *N*_SSA>0.2μm_ was on average for the three bags 1.8 (±0.16) times that of the *N*_SSA>0.2μm_ nighttime minimum (Figures 3a and 4). In the mixed bloom phase, there is an increase in the overall *N*_SSA>0.2μm_ and a more pronounced daytime-to-nighttime variability, with the *N*_SSA>0.2μm_ daytime maximum being 3.9 (±2.3) times higher than the nighttime minimum. During the *G. huxleyi* bloom phase, there is a decrease in the maximum daytime *N*_SSA>0.2μm_ and at nighttime, as *N*_SSA>0.2μm_ steadily decreased, a more drastic drop in *N*_SSA>0.2μm_ appeared about an hour after the PAR decreased to zero (Figure 3). This, in turn, increased the daytime maximum to nighttime minimum *N*_SSA>0.2μm_ ratio to 17.6 (±41.8). Finally, in the *G. huxleyi* demise phase, there was a further decrease in the maximum daytime *N*_SSA>0.2μm_ and a decrease in the daytime-to-nighttime ratio to 2.2 (±3.2).

### Dependence of Daytime SSA Concentration on Biological and Physical Properties of Water

To investigate the influence of various biological and physical water properties on *N*_SSA>0.2μm_, we monitored, once a day, the phytoplankton (calcified *G. huxleyi*, nanophytoplankton, and picophytoplankton), bacterial and ciliate cell counts, the water temperature and salinity, the amount of EhV, chlorophyll a, organic carbon in the form of transparent exopolymer particles (TEP) and particulate organic carbon (POC), and the particulate inorganic carbon (PIC) concentration.^46^ While these daily measurements are insufficient to disentangle the diurnal concentration changes, they help to understand what modulates the SSA emissions as a function of the *G. huxleyi* bloom succession. To evaluate which of these parameters are best correlated with the *N*_SSA>0.2μm_, we calculated the mean *N*_SSA>0.2μm_ between 07:00 and 10:00 (since the water measurements were done around 08:00) each day and looked at its correlation with respect to the 12 measured water properties (Figures 5 and S1). We found that the TEP and POC concentration and bacteria abundance had moderate correlations with the *N*_SSA>0.2μm_ (*R* ^2^ = 0.52; *p* = 0.009, *R* ^2^ = 0.48; *p* = 3 × 10^–4^, and *R* ^2^ = 0.47; *p* = 3 × 10^–11^, respectively. Figure 5A–C, and while the picophytoplankton concentration had moderate correlation with the *N*_SSA>0.2μm_ (*R* ^2^ = 0.41; *p* = 9.8 × 10^–7^), the nanophytoplankton concentration had no correlation with the *N*_SSA>0.2μm_ (Figure 4C,D), which suggests selectivity on how algal species affect *N*_SSA_. Figure S1 shows the other variables, which show no correlation to *N*_SSA>0.2μm_ (*R* ^2^ ≤ 0.35). We also took the photosynthetically available radiation (PAR) measurements from the weather station located on site and could not find a significant relationship between the PAR intensity and the *N*_SSA>0.2μm_ (*R* ^2^ = 0.21; *p* = 0.06; see Figure S2).

**Figure 5:**
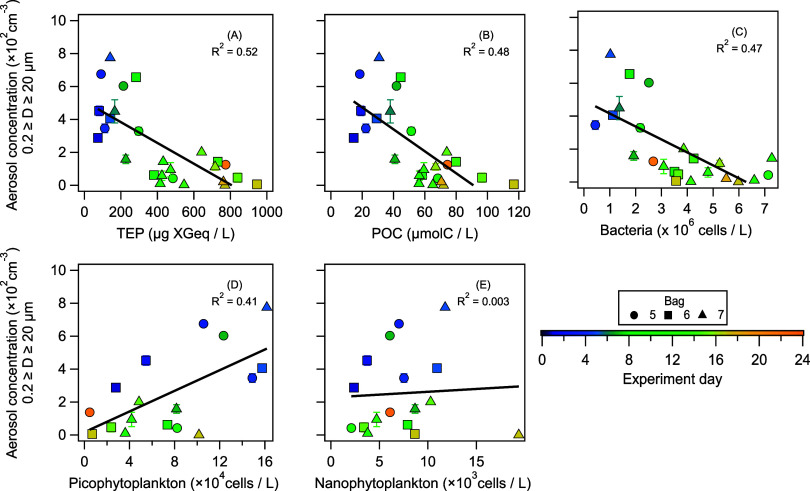
Sea spray aerosol number concentration with diameters *D* ≥ 0.2 μm vs water properties: The dependence of *N*_SSA>0.2μm_ on (A) TEP concentration (*R* ^2^ = 0.52; *p* = 0.009), (B) POC concentration (*R* ^2^ = 0.48; *p* = 3 × 10^–4^), and (C) bacteria abundance (*R* ^2^ = 0.47; *p* = 3 × 10^–11^) shows an anticorrelation, suggesting accumulation of surfactants suppresses SSA emission. While a positive correlation with (D) picophytoplankton concentration (*R* ^2^ = 0.41; *p* = 9.8 × 10^–7^) and the lack of correlation (*R* ^2^ = 0.003; *p* = 1.1 × 10^–7^) with (E) nanophytoplankton suggests selectivity on how algal species affect SSA production. The black line represents the linear fit based on measurements from bags 5, 6, and 7.

## Discussion

Here we followed SSA emission throughout two successive algal blooms in three mesocosm airtight enclosures (Figure 1). The mesocosm enclosures first underwent a bloom of a mixed algal community, consisting mainly of pico- and nanoeukaryotes, followed by a bloom of the coccolithophore *G. huxleyi*, and its consequent demise (Figure 3). We found two concurrent patterns in *N*_SSA>0.2μm_ throughout the algal bloom succession: a diurnal cycle of *N*_SSA>0.2μm_ with higher *N*_SSA>0.2μm_ at daytime, and a decrease in *N*_SSA>0.2μm_ throughout the phytoplankton bloom succession, peaking in the mixed bloom phase and decreasing during the *G. huxleyi* bloom and its demise. These concomitant patterns suggest there are two parallel mechanisms controlling SSA emissions.

What can cause the diurnal cycle of *N*_SSA>0.2μm_? Given the transparent covers placed over the different bags, atmospheric variables such as wind speed or changes in the atmospheric boundary layer height cannot explain the diurnal *N*_SSA_ changes. Additionally, the covers will limit any UV-driven photochemistry on the surface, which can produce sunlight-mediated surfactants, as suggested by Long et al.^13^ While the covers are 90% permeable to PAR, they block UV radiation. Typical daily values in this region during May and June vary between 38.5 and 41.8 Einstein m^–2^ day^–1^, while in the open ocean range from ∼10–55 Einstein m^–2^ day^–1^ (Figure S3), depending on latitude. The PAR values during the experiment were higher than the seasonal average (Figure S3). Neither oceanic variables, such as water temperature, salinity, and chlorophyll-*a* concentration, can explain the diurnal *N*_SSA_ changes. Water temperature and chlorophyll-*a* concentration had similar behavior during the experiment: gradually increasing during the initial and mixed bloom stages, then declining during the first days of the *G. huxleyi* bloom, followed by a second increase, to finally have a steady decline in the *G. huxleyi* decline phase (Figure S4). Salinity remained almost constant around 30.85 (±0.05) g/kg up to day 20, with daily changes of less than 0.1 g/kg. If water temperature, salinity or chlorophyll-*a* were controlling the diurnal *N*_SSA_ changes, we would expect the nighttime *N*_SSA_ to follow a similar pattern during the experiment.

Since we observed the diurnal emission change for all measured SSA diameters (Figures 2 and 3), this suggests there is a parallel mechanism to the induced bubble-bursting in each bag that either creates more bubbles and/or changes the film properties (lifetime and thickness). Given the diurnal pattern of the emitted SSA, we hypothesize the response originates from light-dependent biological processes. We conjecture that the diurnal pattern is related to photosynthesis byproducts that can affect the quantity of bursting bubbles. The increased number of bubbles could occur during photosynthesis, as phytoplankton produce oxygen, and the changes in oxygen concentration in the water may increase bubble production. This will not only affect the *N*_SSA>0.2μm_, but also SSA with diameters less than 0.2 μm. Within the ocean’s surface, it was previously shown that bubble growth could occur below supersaturation,^56^ and as the level of dissolved oxygen increases (above 100%) there is an increase in the production of bubbles^57^ and droplets (i.e., SSA).^58^ In the bags, due to the continuous mixing caused by air entrainment, the oxygen concentration will probably be at or near supersaturation, and any oxygen release due to photosynthesis will consequently increase this supersaturation. This could explain the initial increase in *N*_SSA>0.2μm_ in the mixed phase part of the experiment, where nano- and picophytoplankton increase rapidly, together with the greater number of bacteria (Figures 5C,D and 2). In the open ocean surface, the conditions for diurnal SSA production are favorable: oxygen saturation levels mostly exceed 100%,^59–61^ with biologically induced (net photosynthetic production) O_2_ supersaturation.^62–64^ Measurements of dissolved oxygen from shallow near-shore water showed oxygen supersaturation levels high enough to cause bubble growth within the upper 1.5 m.^56,57^ The process can be further enhanced by oxygen from entrained bubbles by breaking waves,^65,66^ even in supersaturated conditions.^67^ However, the only (indirect) evidence of this effect on *N*_SSA_ might be the recent observation, with in situ measurements, of diel *N*_SSA_ patterns along the Pacific and Atlantic Oceans and the Caribbean Sea.^15^ The highest day-to-night differences of *N*_SSA_ in oligotrophic waters were detected where the influence of long-range transported aerosols was minimal, as these can mask the diurnal signal. On the other hand, in water with a salinity of less than 7.1 g/kg, a link between decreasing aerosol production during the day and dissolved oxygen concentration was observed.^44^ Other studies investigating SSA production differences between day and night by generating SSA artificially from seawater at sea, observed an increase in SSA number and mass during the day in biologically active waters, but not in oligotrophic ones.^13,14^ They attributed this increase to biogenic surfactants released by marine biota or formed photochemically.

Concomitant to the diurnal effect, what can be causing the suppression of *N*_SSA>0.2μm_ during the *G. huxleyi* bloom and its demise? This suppression could be the result of the accumulation of surfactants from the phytoplankton,^68^ which can counteract the possible increased bubble production from nano- and picophytoplankton and bacteria. We observe this by the anticorrelation of *N*_SSA>0.2μm_ with the TEP and POC concentrations (Figure 5A,B) and the increase in TEP and POC concentration throughout the experiment (Figure 3F and Vincent et al.^46^). TEPs are a special class of exopolymers, gel-like surface-active particles that are secreted by many marine microorganisms.^40^ TEPs can rise to the surface in the open ocean to form or stabilize the sea surface microlayer.^41^ Different algal species exhibit different TEP production rates,^69^ and while the chemical composition of TEP remains largely uncharacterized,^40,70^ it is likely that each species produces distinct polymers that form TEP.^71^ Here, TEP production is strongly associated with *G. huxleyi* abundance and viral infection (ref 46 and Figure S5). While picophytoplankton, nanophytoplankton, and bacteria drive production before the *G. huxleyi* bloom (Figure S5). This, together with the low to no correlations of *N*_SSA>0.2μm_ with *G. huxleyi* and EhV abundance (Figure S1), suggests that it is the accumulation of TEP together with the release of other surface-active compounds from POC in the water that reduces *N*_SSA>0.2μm_. While there is also an anticorrelation of *N*_SSA>0.2μm_ with bacteria abundance, it does not explain the decrease in *N*_SSA>0.2μm_. On the one hand, bacteria had a maximum abundance at day 11, followed by lower concentrations thereafter; on the other hand, it has been previously shown that it is the secretions and not the presence of bacteria that change bubble bursting dynamics.^72^

The different day-to-night *N*_SSA>0.2μm_ ratios throughout the experiment suggest a differential, species-specific influence on SSA production in algal blooms. The increased amount of surfactants has been shown to decrease SSA production^30,35,73^ by lowering the surface tension and, in turn, reducing droplet production.^13^ The decrease in *N*_SSA_ may influence SSA production in high latitudes as *G. huxleyi* is driven polewards^74,75^ due to climate change. While recent evidence suggests a significant increase in SSA production in colder temperatures,^30^ this could be dampened by the decrease we observed here when *G. huxleyi* is at its bloom stage or in its demise, as virus-infected cells can produce 4.5-fold more TEP.^46,50^

The diurnal and weekly variations in emitted *N*_SSA_ during different stages of the *G. huxleyi* bloom underscore the dynamic interplay between daily and accumulated biophysical changes. Furthermore, our observations suggest that diurnal variability in SSA emissions may be more common than previously appreciated. If widespread, it has important implications for air–sea exchange processes, marine aerosol radiative forcing, and cloud microphysical properties. A diel rhythm in *N*_SSA_ introduces systematic variability in aerosol number and size distributions over daily time scales. This could, in turn, affect cloud condensation nuclei availability, which could affect droplet number concentration and cloud reflectivity in marine boundary layers. Current models assume equal day and nighttime SSA emissions, missing subdaily variability. Incorporating such temporal dynamics may improve the accuracy of short-term weather forecasts and longer-term climate projections. This emphasizes the need for further research on the impact of biological activity at the ocean surface on emitted sea spray aerosols.

## Supplementary Material



## Data Availability

All water measurements are available in Vincent et al.^46^ The aerosol data set is available from the corresponding author upon reasonable request.

## Notes

Additional figures showing correlations between sea spray aerosol concentrations and biological, chemical, and physical parameters in the water; photosynthetically available radiation data; temporal patterns of water temperature, salinity, and chlorophyll-a concentration during the experiment; and relationships of transparent exopolymer particles with microbial variables (PDF)
