# Supplementary material for: Diurnal Emissions of Sea Spray Aerosols in Algal Blooms
Source: Environ Sci Technol. 2025 Nov 3;59(45):24463–72. doi: 10.1021/acs.est.5c12650 (PMC12643528; doi:10.1021/acs.est.5c12650)
Supplement: Supplementary file 1 [file es5c12650_si_001.pdf]

Supplementary information

Diurnal emissions of sea spray aerosols in algal blooms

J. Michel Flores[1], Miri Trainic[1], Daniella Schatz[2], Ilan Koren[1], Assaf Vardi[2]
[1]Weizmann Institute of Science, Department of Earth and Planetary Science, Rehovot, Israel
[2]Weizmann Institute of Science, Department of Plant and Environmental Sciences, Rehovot, Israel

The supporting information file contains six pages with additional figures showing correlations between sea spray aerosol concentrations and biological, chemical, and physical parameters in the water (Fig. S1); photosynthetically available radiation data (Fig. S2 and Fig. S3); temporal patterns of water temperature, salinity, and chlorophyll-a concentration during the experiment (Fig. S4); and relationships of transparent exopolymer particles with microbial variables (Fig. S5).

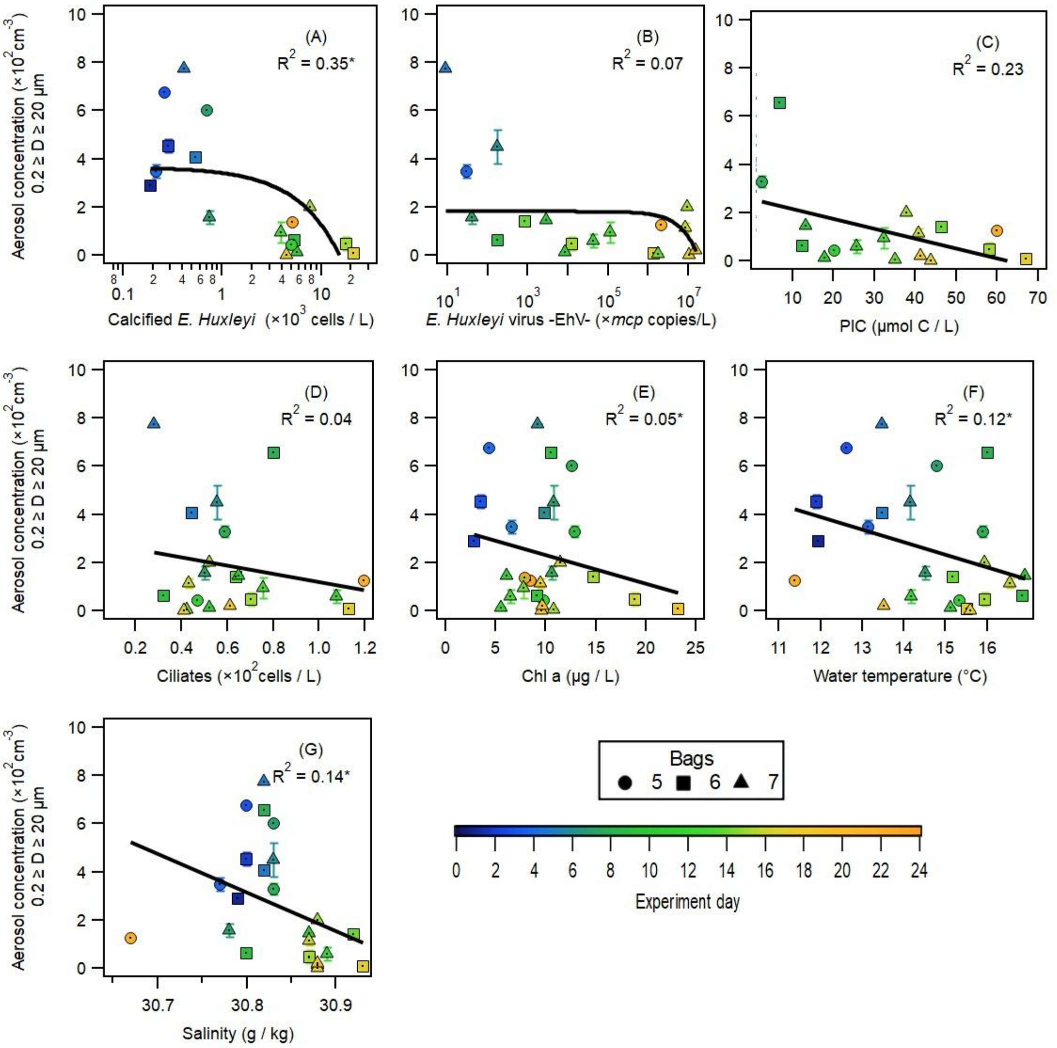

Figure S1. Dependence of the sea spray aerosol number concentration with diameters D ≥ 0.2 µm on: (A) calcified *E. huxleyi* abundance, (B) *E. huxleyi* virus (EhV) concentration, (C) particulate inorganic carbon (PIC), (D) abundance of ciliates, (E) chlorophyll a concentration, (F) water temperature, and (G) salinity. The black line shows the linear fit from all the measurements of bags 5, 6, and 7. Asterisks (*) indicate significant correlations (linear model, $p < 0.01$).

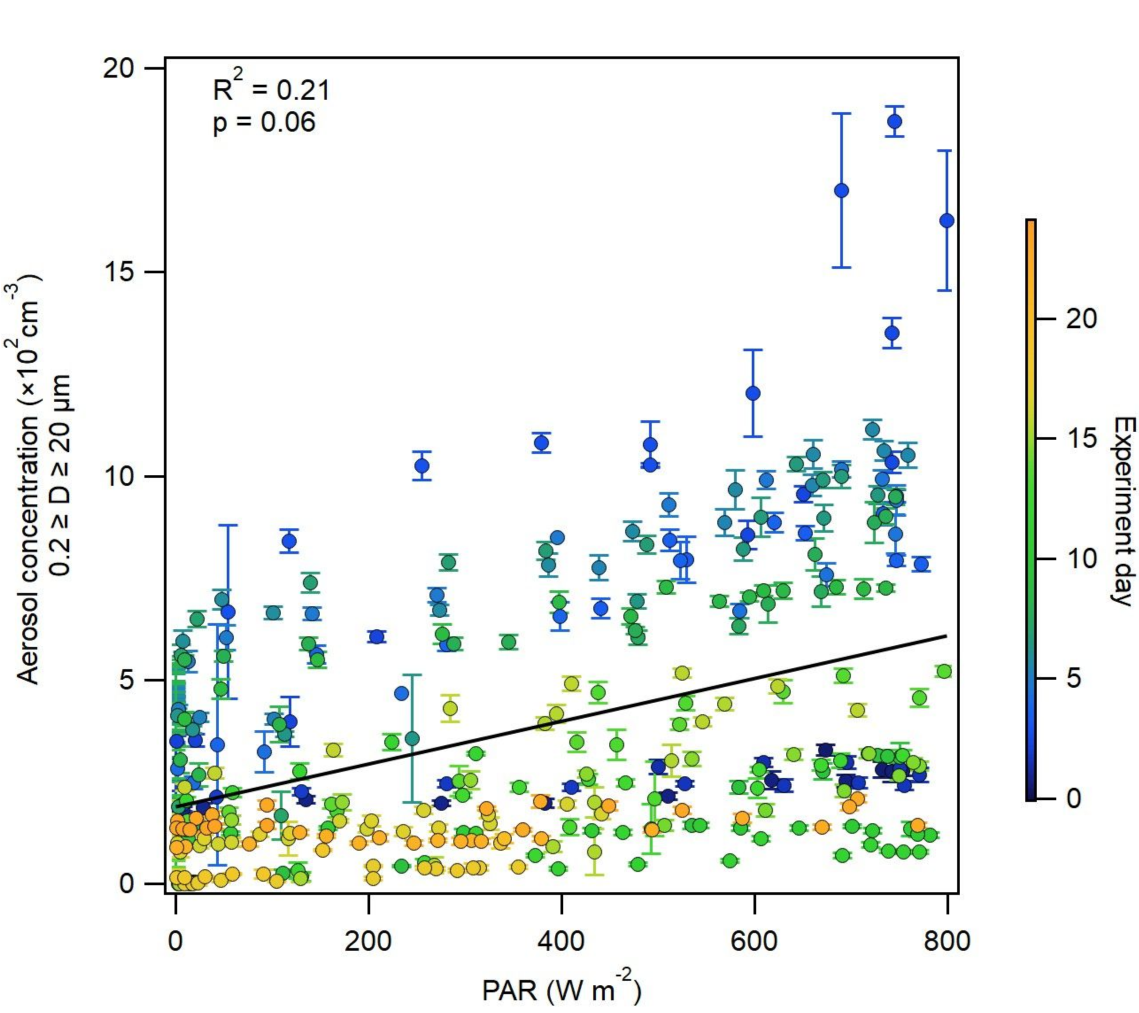

Figure S2. Dependence of the sea spray aerosol number concentration with diameters D ≥ 0.2 µm on the photosynthetic available radiation (PAR) during the experiment.

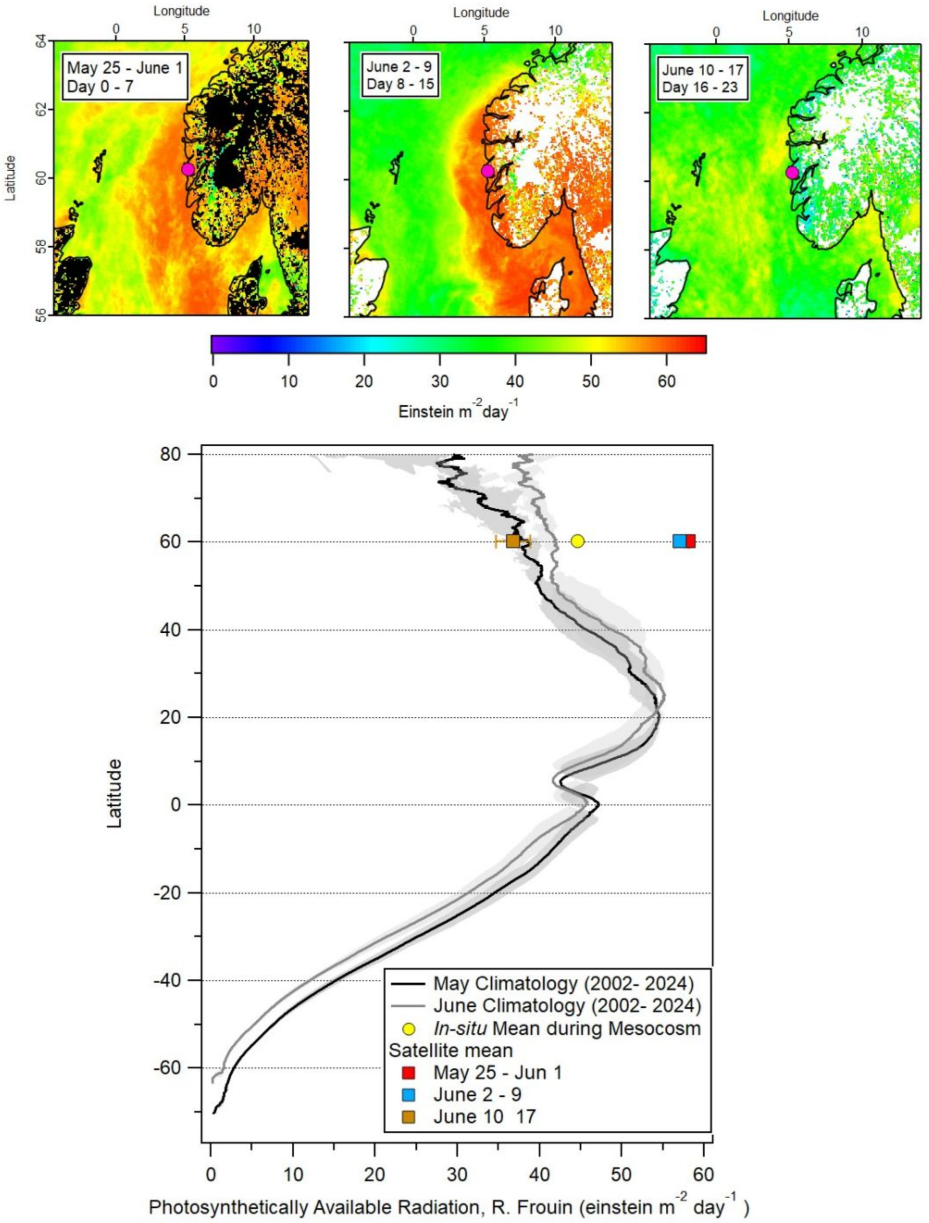

Figure S3. **Mean photosynthetically available radiation (PAR) during the mesocosm experiment.** Top panels: 8-day composites of PAR from Aqua-MODIS (4 km resolution, Level 3 data). The pink circle indicates the location of the marine station. Lower panel: Comparison between the *in-situ* mean PAR value during the mesocosm experiment (yellow circle), the 22-year climatological zonal means of May (black line) and June (grey line), and the mean values from the 8-day composites for day 0 to 7 (red square), day 8 to 15 (blue square), and day 16 to 23 (orange square). The zonal means were calculated using Aqua-MODIS Level 3 PAR data (4 km resolution) from May and June (2003–2025). The mean *in-situ* PAR was derived from meteorological data collected at the marine station. The satellite composite means were calculated by averaging a 3 × 3 pixel box centered on the station's coordinates (60°16′11″N, 5°13′07″E), covering the area from 60°12′N to 60°18′N and 5°09.6′E to 5°15′E.

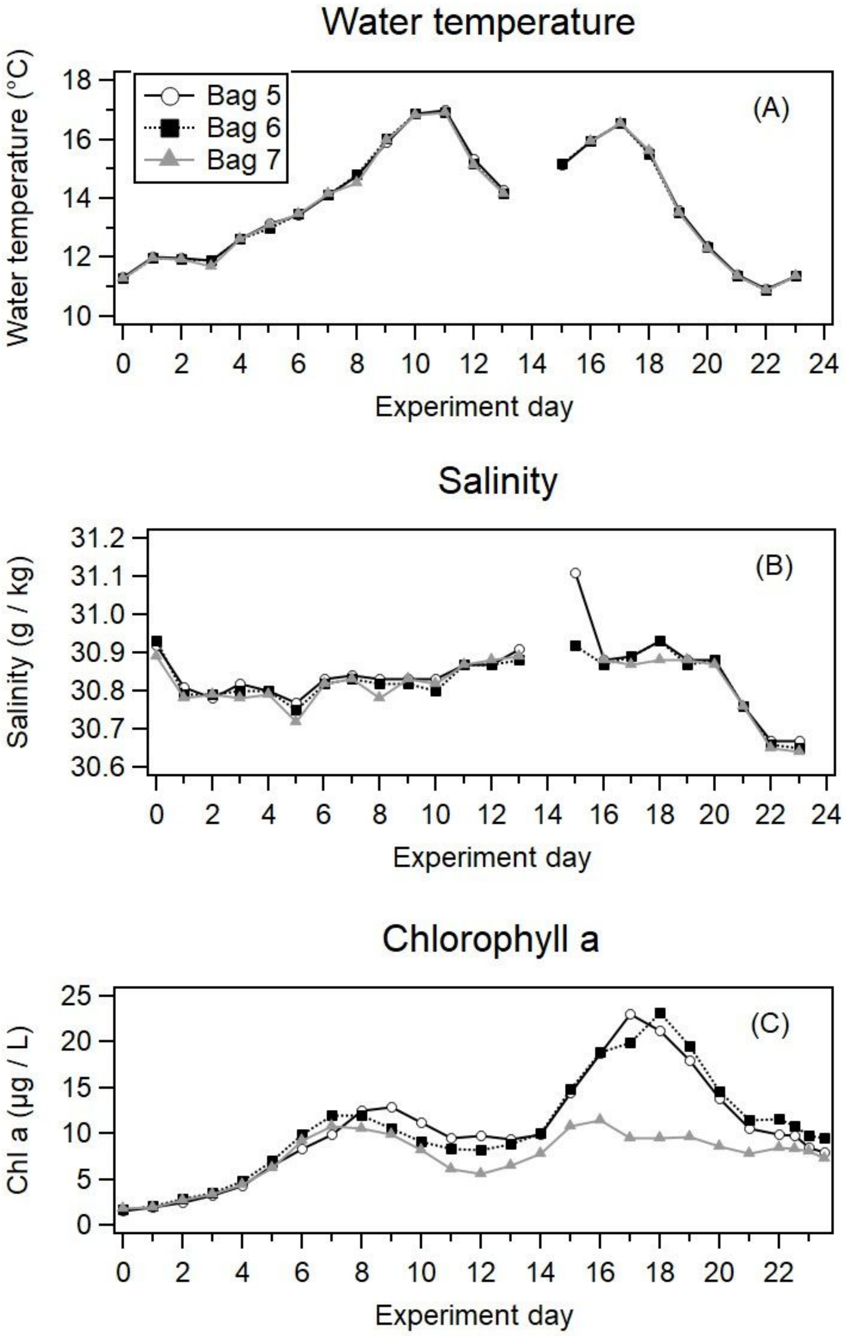

Figure S4. Temporal patterns of (A) water temperature, (B) salinity, and (C) chlorophyll-a concentration during the experiment.

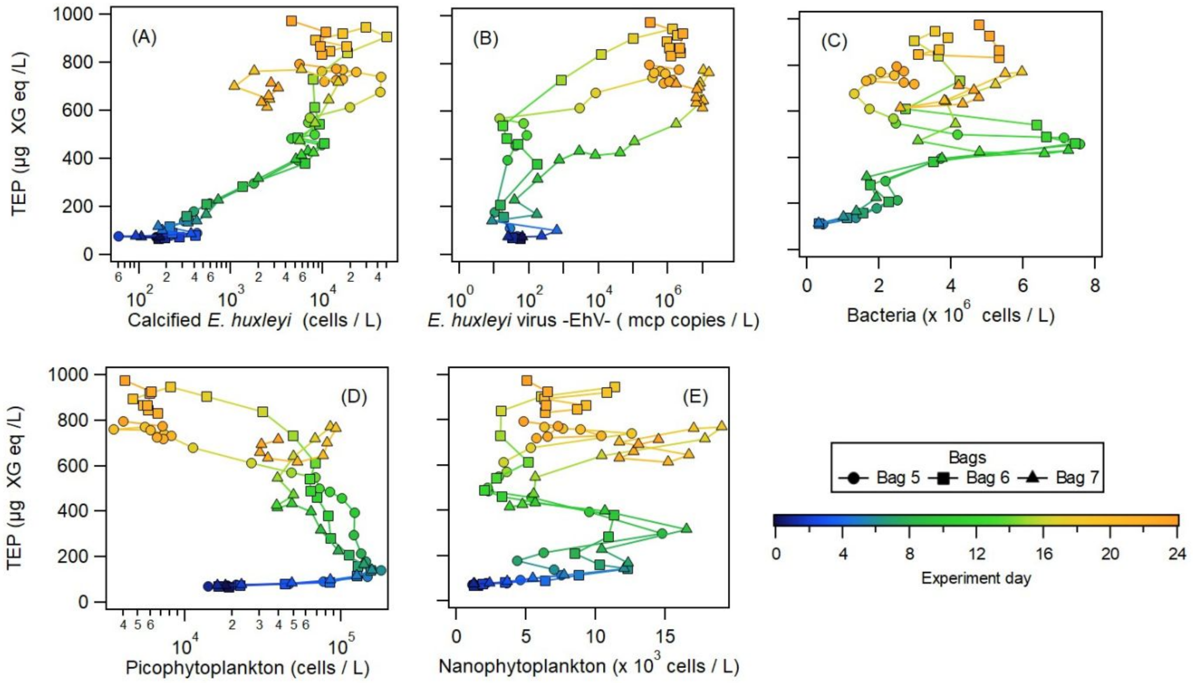

Figure S5. Dependence of the Transparent exopolymer particles (TEP) on: (A) calcified *E. huxleyi* abundance, (B) *E. huxleyi* virus (EhV) concentration, (C) bacteria abundance, (D) picophytoplankton and (E) nanophytoplankton concentration.
